# Dr. Kinglake, on Digitalis

**Published:** 1804-01-01

**Authors:** Robert Kinglake

**Affiliations:** Taunton


### *To the Editors of the Medical and Physical Journal.*

GENTLEMEN,

THE advocates for medical improvement, cannot but lament that the well-earned reputation of *digitalis*, for its general remedial power in the early, or curable, stages of pulmonary consumption, should be called in question by an experience, much too limitted to justify even a suspicion, much less an unqualified attack on its admitted efficacy.

My allusion is to the *two cases* detailed by Mr. Earnest, in the last number of your Journal,* in which, that very active medicine is stated to have been almost inert, or at

---

* Page 551—7.

least least to have had no salutary influence whatever. The tincture of digitalis, as proposed by the judicious Dr. Maclean, was the preparation employed, which tincture appears to possess the virtues of the plant in the utmost degree of concentration. The medicine then, in a vast majority of cases, must have been impressively active; and, agreeably to the observation of others, in no small proportion beneficially so. Mr. Earnest's cases, therefore, do not fairly impeach the salutary properties of the medicine, but denote only, that either an unexplained peculiarity, or a positive incurability, placed the work of relief out of the reach either of that or any other remedy. In what morbid relations the cases stood with the acknowledged efficacy of digitalis, is not stated, but might have been somewhat elucidated by an examination of the lungs after death.

It has scarcely been pretended, that digitalis ever cured the last stage of pulmonary consumption; it would savour too much of the daring declamation of quackery, to insist that such a necessarily, deadly, and actually dying state of life, could possibly be restored to health.

If the beneficial agency of a medicine be determinable by its occasional failure, not an article in the materia medica would abide such an estimate of its virtue. Even Peruvian bark, opium, mercury, and antimony, would shrink from such a test of infallibility.

Digitalis has deservedly taken its station in the list of the most active remedies; nor can it be degraded from its just rank by an inadequate trial of its powers. After what has been affirmed of its salutary influence in pulmonary consumption, *two hundred,* instead of *two cases,* should have been pressed into the service of its opposition, to have warranted assailing its high credit. Nor would my scepticism in its inefficacy have been persuaded, even on the authority of two hundred adverse cases, if the trial had not been extended beyond three weeks.

A much longer period is necessary to perform the mighty work of curing pulmonary consumption; and, indeed, when that threatening disorder is curable, it has been found that the best efforts of digitalis require to be urged, and sustained, from at least one month to three, to insure a permanent recovery.

Peculiar and inscrutable irrelevancy of temperament to specific medicinal efficiency, does not preclude the possibility of its being highly influential in more congenial circumstances of excitability; it amounts to nothing more than than an exception to a general rule, and is of no practical authority.

The experienced observer of the salutary agency of digitalis in pulmonary consumption, has not to learn that cases might, and do occur, in which that medicine is of no avail in that disease ; but these instances are so abundantly counterbalanced by its successful operation, as to reject them from all calculation, to the disparagement of its real efficacy.

My own experience has furnished me with many instances, in which digitalis was either inert, or manifestly injurious; but these presented amidst a groupe of salutary efficiency, that enabled me to assign to the medicine, a degree of curative power, which none equal in pulmonary consumption, and but few exceed in any other disorder.

Mr. Earnest's humanity, science, and zeal for medical improvement, should induce him to rejudge his cases, and suspend his inference to the disadvantage of digitalis, until a research, commensurate with the importance of the subject, and the opportunities his public situation (as house surgeon to the Sheffield Infirmary) so amply affords him, shall authorise him to deliver an unequivocal and decisive opinion on its anti-phthisical virtue.

The average result of one hundred trials, conducted with Mr. Earnest's apparent accuracy, but extended, if necessary, to three months duration, will certainly, if any faith is due to experience, at once justify this comment, and convince Mr. Earnest, that digitalts has an irresistible claim to confidence for *generally* curing the early, and alleviating the late, stages of pulmonary consumption.

*Taunton,*
*Dec.* 10, 1803.

